# Supplementary material for: Predicting visual function by interpreting a neuronal wiring diagram
Source: Nature. 2024 Oct 2;634(8032):113–23. doi: 10.1038/s41586-024-07953-5 (PMC11446822; doi:10.1038/s41586-024-07953-5)
Supplement: Supplementary file 6 — CRF and ERF predictions for individual TmY4 and TmY9 cells. Analogous to Supplementary Data 3, but for TmY target types. Shown are the top four monosynaptic pathways, the strongest pathway passing through each of the top ten intermediary types (ranking from Extended Data Fig. 7), and the trisynaptic pathway Tm1–TmY–Dm3–TmY (see the section entitled Prediction of spatial normalization). [file 41586_2024_7953_MOESM6_ESM.zip › DataS4/TmY9qΓèÑ/720575940614967106.pdf]

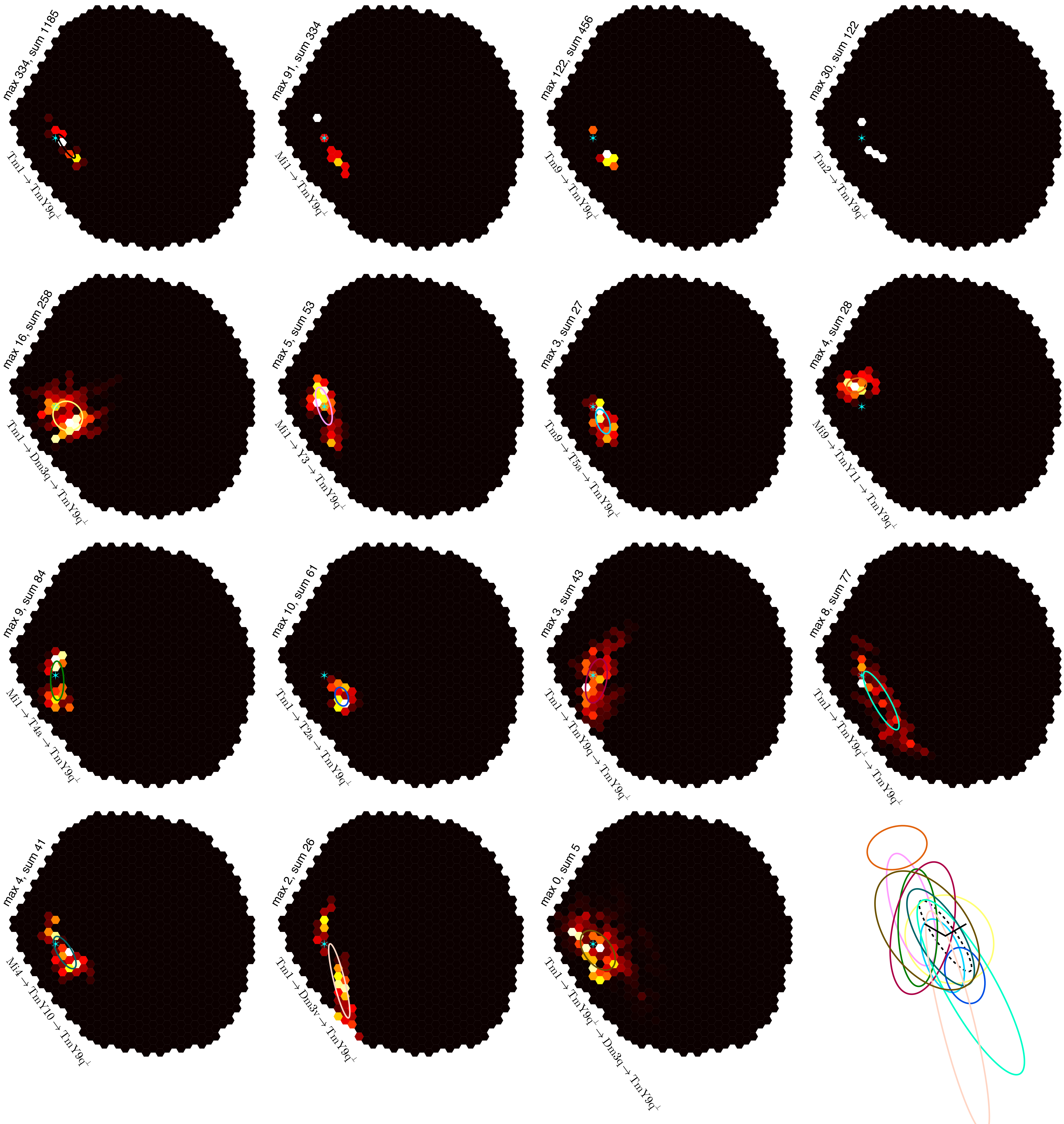
max 334, sum 1185
Tm1 → TmY9q$^{\perp}$
max 91, sum 334
Mi1 → TmY9q$^{\perp}$
max 122, sum 456
Tm9 → TmY9q$^{\perp}$
max 30, sum 122
Tm2 → TmY9q$^{\perp}$
max 16, sum 258
Tm1 → Dm3q → TmY9q$^{\perp}$
max 5, sum 53
Mi1 → Y3 → TmY9q$^{\perp}$
max 3, sum 27
Tm9 → T5a → TmY9q$^{\perp}$
max 4, sum 28
Mi9 → TmY11 → TmY9q$^{\perp}$
max 9, sum 84
Mi1 → T4a → TmY9q$^{\perp}$
max 10, sum 61
Tm1 → T2a → TmY9q$^{\perp}$
max 3, sum 43
Tm1 → TmY9q → TmY9q$^{\perp}$
max 8, sum 77
Tm1 → TmY9q$^{\perp}$ → TmY9q$^{\perp}$
max 4, sum 41
Mi4 → TmY10 → TmY9q$^{\perp}$
max 2, sum 26
Tm1 → Dm3v → TmY9q$^{\perp}$
max 0, sum 5
Tm1 → TmY9q$^{\perp}$ → Dm3q → TmY9q$^{\perp}$
